# Per-Pixel Coded Exposure for High-Speed and High-Resolution Imaging Using a Digital Micromirror Device Camera

**DOI:** 10.3390/s16030331

**Published:** 2016-03-04

**Authors:** Wei Feng, Fumin Zhang, Xinghua Qu, Shiwei Zheng

**Affiliations:** State Key Laboratory of Precision Measuring Technology and Instruments, Tianjin University, Tianjin 300072, China; david0806@tju.edu.cn (W.F.); quxinghua@tju.edu.cn (X.Q.); shiweizheng@tju.edu.cn (S.Z.)

**Keywords:** high-speed imaging, DMD camera, per-pixel coded exposure, quicksort

## Abstract

High-speed photography is an important tool for studying rapid physical phenomena. However, low-frame-rate CCD (charge coupled device) or CMOS (complementary metal oxide semiconductor) camera cannot effectively capture the rapid phenomena with high-speed and high-resolution. In this paper, we incorporate the hardware restrictions of existing image sensors, design the sampling functions, and implement a hardware prototype with a digital micromirror device (DMD) camera in which spatial and temporal information can be flexibly modulated. Combined with the optical model of DMD camera, we theoretically analyze the per-pixel coded exposure and propose a three-element median quicksort method to increase the temporal resolution of the imaging system. Theoretically, this approach can rapidly increase the temporal resolution several, or even hundreds, of times without increasing bandwidth requirements of the camera. We demonstrate the effectiveness of our method via extensive examples and achieve 100 fps (frames per second) gain in temporal resolution by using a 25 fps camera.

## 1. Introduction

High-speed photography is an important tool for studying rapid physical phenomena in shock waves, fluidics, collision, photochemistry, and photophysics [1,2,3,4,5]. The CCD or CMOS sensor has been widely used in optical imaging technology by far. They have several advantages, such as small size, high quality image, relatively low cost, and digital imaging. However, off-the-shelf CCD or CMOS cameras usually have a frame rate of 30 Hz, and cannot effectively capture the rapid phenomena with high-speed and high-resolution. The main technological limitation is that it takes time for the data readout and storage from the sensor array.

To address the bandwidth limitation for scientific research and industrial applications, various high-speed imaging methods have been developed and commercialized over recent decades. In general, the conventional high-speed imaging methods for solid-state image sensors can be classified into two categories. One is the use of parallel readout imaging scheme [6,7,8]. Tochigi *et al.* had demonstrated a global-shutter high-speed CMOS image sensor with a readout speed of 1-Tpixel/s in burst video and 780-Mpixel/s continuous [9]. It is noteworthy that the more readout ports, the more complex it is for the drive circuit. Currently, imaging frame rate based on parallel readouts method cannot reach 10^5^–10^7^ fps. The other breakthrough method is ISIS (*In situ* Storage Imaging Sensor), which was introduced for the development of a video camera capable of more than 10^6^ fps. The key to this technology is the image sensor chip production, and its essence is the use of parallel readout of all pixels and pixel transfer and storage. In 1996, Lowrance had used a burst image sensor with “SPS (Serial-Parallel-Serial) storage” concept for the first time to develop a max frame rate of 10^6^ fps with 32 consecutive frames [10]. Then the ISIS technology has experienced rapid development, from the most primitive hole-type, linear-type to the slanted linear image sensor *in situ* storage [11,12,13,14,15]. Recently, Etoh *et al.* had developed a backside illuminated (BSI) CCD image sensor, mounting the ISIS structure and placing metal wires on the front surface to distribute driving voltages of the *in situ* CCD memory, and its highest frame rate can reach 16 Mfps with higher sensitivity [16]. The image sensor performances, including resolution, frame rate, frame continuous shooting and so on have been greatly improved. Nevertheless, the ISIS needs to complete the data readout and storage in a very short frame interval, and its sensitivity is insufficient due to the lack of light throughput.

Therefore, the conventional high-speed imaging methods require a solid-state image sensor with high photoresponsivity at a very short integration time, synchronous exposure, interframe coherence, and high-speed parallel readout due to the necessary bandwidth. In addition, they are also faced with massive data storage problems in real time. Because of all these inherent bandwidth limitations, it is difficult to achieve a higher imaging frequency, larger space bandwidth, and higher frames.

Can we turn a low-frame-rate camera into a powerful high-speed video camera for capturing the rapid phenomena with high-speed and high-resolution without increasing bandwidth requirements? With the advent of computational cameras, a new approach to achieve high-speed imaging is offered. Computational cameras combine the features of the computer and the camera, it samples the light field in a completely different way to create new and useful forms of visual information [17]. In recent years, many scientists in this way make some beneficial attempts. Raskar *et al.* had built a coded-exposure camera with a ferroelectric liquid crystal shutter to address motion blur, and the camera shutter was controlled opened and closed during the chosen exposure time with a binary pseudo-random sequence [18]. Gupta *et al.* demonstrated a co-located camera-projector system that could enable fast per-pixel temporal modulation [19]. Olivas *et al.* had proposed a computational imaging system based on position sensing detector (PSD) to reconstruct images degraded by spatially variant platforms in low-light conditions [20]. In order to perform temporal super resolution, Reddy *et al.* had designed a reconstruction algorithm that used the data from a programmable pixel compressive camera (P2C2) along with additional priors [21]. Liu *et al.* had proposed techniques for sampling, representing, and reconstructing the space-time volume by using a prototype imaging system based on a liquid crystal on silicon device [22]. Veeraraghavan *et al.* had presented a sampling scheme and reconstruction algorithm, and turned an off-the-shelf camera into a high-speed camera for periodic signals based on coded strobing photography [23]. In addition to the ideas proposed above, Bub *et al.* had proposed a temporal pixel multiplexing (TPM) paradigm to realize simultaneous high-speed and high-resolution imaging [24]. In Bub *et al.*’s work, an imaging modality by using a DMD could flexibly control the trade-off between spatial and temporal resolution, and it was implemented to offset pixel-exposure times during a frame time of the camera. However, in the imaging modality they propose, the TPM, as far as we can tell, is just a paradigm, which has not been theorized. Meanwhile, the image processing algorithm is relatively complex for the coded exposure image. All those factors would limit the application of the temporal pixel multiplexing.

Instead of increasing bandwidth requirements, we exploit a computational camera approach based on per-pixel coded exposure to achieve high-speed imaging. With the flexible controller and advanced micro-electromechanical system (MEMS) techniques, the DMD mirror direction can be controlled by digital signals within 24 μs [25], which is approximately 1/1000th of the frame time of an ordinary CCD camera. Therefore, the per-pixel coded DMD pattern can be easily flipped by many times during one integration time of the CCD camera, and the temporal information can be embedded in each frame by modulating light. After that, different subframes can be extracted based on the light intensity distribution of the image. As a result, the computational camera can simultaneously acquire high-speed and high-resolution images compared with a conventional digital camera.

The per-pixel coded exposure method has several advantages compared with traditional high-speed imaging methods. Thus, it becomes possible that the low-frame-rate cameras with their known advantages in terms of low cost, signal-to-noise ratio (SNR), and high dynamic range can be also used for high-speed imaging while without increasing the inherent properties of the image sensor. The light intensity amplitude of imaging system should be kept constant. In this case, static regions of the full-resolution image are unchanged at native detector frame, and high-speed image sequences are obtained by temporal information at reduced resolution. Therefore, this approach not only used one detector to achieve high-speed and high-resolution imaging, but also did so without increasing the bandwidth requirements for data transfer and storage which are the same as the original single detector. Moreover, this approach uses a new programmable imaging system, and it also supports different spatial light modulators or different frame rates for the different areas of the image sensor, which may be useful for unconventional detectors.

In addition, the pixel binning is a commonly known technique to increase frame rate and SNR at the sacrifice of spatial resolution [26], which may be similar to the per-pixel coded exposure method. However, there are fundamental differences between them. On the one hand, the pixel binning is the process of combining the electrical charge from adjacent multiple pixels together to form a new pixel, and the charge amount of the new pixels may easily exceed the capacity of the CCD potential well, which will cause over-saturation and result in information distortion. On the other hand, the pixel binning can only use the rectangular masks such as 2 × 2 binning, 3 × 3 binning to form one single large pixel, which is a lack of flexibility. By contrast, the per-pixel coded exposure method can realize simultaneous high-speed and high-resolution imaging and does not easily cause over-saturation. Furthermore, the DMD masks can be represented by binary encoding, which may have different shapes, including diamond-shaped, square, rectangular, and other nonrectangular regions of interest. Therefore, the per-pixel coded exposure method is more flexible and adaptable compared with the pixel binning method.

In this paper, we incorporate the hardware restrictions of existing image sensors, design the sampling functions, and implement a hardware prototype with a DMD camera in which spatial and temporal information can be flexibly modulated. We build and analyze the optical model of the DMD camera, then theoretically analyze the per-pixel coded exposure and propose a three-element median quicksort method to increase the temporal resolution of imaging system. The rest of this paper is organized as follows: firstly, the outline and the optical model of our DMD camera are introduced in Section 2. Then the theory of per-pixel coded exposure for high-speed imaging is proposed in Section 3, where the DMD pixel-level modulation process, the sampling clock control and extraction algorithm of subframe are described in detail. Experimental results are given in Section 4. Finally, conclusions are summarized in Section 5.

## 2. Hardware Implementations

In this section, we show the details of our prototype imaging system. Firstly, we describe the deflection characteristics of the DMD mirror and the outline of the DMD camera, respectively. Then, we build and analyze the optical model of the DMD camera. After that, we implement our DMD camera with mirror-to-pixel corresponding adjustment.

### 2.1. DMD Camera

**Figure 1:**
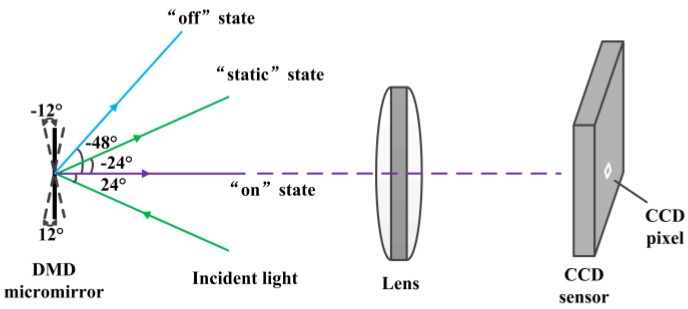
The direction of DMD mirrors.

As a spatial light modulator, DMD consists of hundreds of thousands of micro-mirrors on a chip. All mirrors can be individually modulated from an on-off pattern signal given to the DMD within 24 μs by applying an address voltage. The DMD is able to change the direction of incident light and each mirror represents one pixel of the CCD sensor as shown in Figure 1. The DMD mirror angle is +12°, −12°, or 0 corresponding to an “on”, “off”, or “static” state. When the incident light angle is 24°, the reflection light angle is 0 (“on” state) or −48° (“off” state). The DMD can be seen as an optical digital arithmetic unit, and it has been widely used in bioassays, 3D optical metrology, and photodetectors [27,28,29].

**Figure 2:**
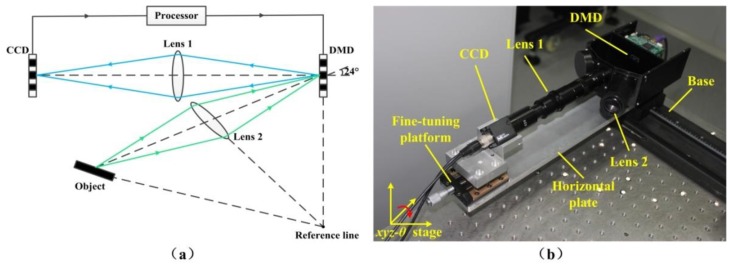
DMD camera. (**a**) Optical schematic of our system; (**b**) Imaging system layout.

Recently, we have developed a DMD camera as shown in Figure 2a. The DMD camera comprises a DMD, a CCD, image processor, and two lenses (lens 1 and lens 2). In terms of system architecture, the positions of DMD, lens 2 and the object should satisfied the well-known scheimpflug condition [30]. That is, when the three planes meet in the reference line, where the positions of the object and the DMD micromirror array are in a conjugate relationship, the object can be ideally imaged on the surface of the DMD by lens 2. The position of DMD and CCD are arranged in face-to-face relation and each DMD mirror corresponds to the CCD pixel on the optical axis of lens 1. The CCD and DMD form the photoelectric feedback structure by the image processor. In terms of working principle of the system, it can be described as follows. First, the reflected light from an object is projected onto the DMD surface by lens 2. Then the DMD image can be reimaged on the CCD camera by lens 1. The DMD is used as a precision optical switch, and it determines whether the reflected light enters the CCD camera or not. When the DMD mirror is set to “on” state, the reflected light coming from the object can be imaged at the CCD camera successively through lens 2 and lens 1. However, when the DMD mirror is set to “off” state, the reflected light cannot be imaged at the CCD camera. In this way, the DMD completes the selective imaging for the spatial incoming light in two-dimensional space.

Figure 2b shows the hardware prototype of DMD camera in our experiment. A CCD camera (Teli CS8620Ci, 768 × 576 pixels, 25 fps) is placed on a fine-tuning platform, which is an adjustable *xyz*-θ stage and can be tilted in four coordinates. The DMD (Texas Instruments 0.3 WVGA 220, 684 × 608 mirrors) and its accessory light modulator package (ALP) are fixed and sealed in a metal shell. The metal shell and the fine-tuning platform are fixed on a horizontal plate to make sure that the optical path of DMD and CCD are coaxial. Lens 1 (zoom lens, a paraxial magnification of 1.12) and lens 2 (100 mm focal length) are used.

### 2.2. Model of Optical System

In the linear space invariant imaging system composed of DMD and CCD [31], a point on the object plane is mapped not only as a corresponding points on the image plane, but also be spread into an area. Therefore, the single point on the image plane is actually the superposition of many corresponding points on the object plane. The superimposing imaging process can be expressed by a superposition integral in mathematics.

(1) $$g(x,y)=\int_{-\infty }^{+\infty }\int_{-\infty }^{+\infty }f(s,t)h(x,y;s,t)dsdt=f(s,t)⊗h(x,y;s,t)$$

where (*s*, *t*) and (*x*, *y*) represent the two-dimensional spatial coordinate point on the object plane and the image plane, respectively; *f*(*s*, *t*) represents an observed object image; *g*(*x*, *y*) represents the image captured by camera, or known as the degraded image; *h*(*x*, *y*; *s*, *t*) described the optical field distribution of the imaging system is called the point spread function (PSF).

The light intensity function *I*(*x*, *y*) can be represented the time average of dot product between the image function *g*(*x*, *y*) and the conjugate image function g* (*x*, *y*).

(2) I(x,y)=〈g(x,y)⋅g*(x,y)〉

Inserting Equation (1) into Equation (2), the light intensity function *I*(*x*, *y*) also can be expressed as

(3) I(x,y)=〈(f(s,t)⊗h(x,y;s,t))⋅(f*(s,t)⊗h*(x,y;s,t))〉=|f(s,t)|2⊗|h(x,y;s,t)|2⊗〈Re{2f*(s,t)h*(x,y;s,t)}〉

Since the reflected light from an object can be seen as a non-coherent plane wave, then the Equation (3) can be further obtained

(4) $$I\left(x,y\right)={\left|f(s,t)\right|}^{2}⊗{\left|h(x,y;s,t)\right|}^{2}$$

Let *I*(*x*, *y*, *t*) denote the space-time volume corresponding to an *M* × *N* pixels neighborhood and one frame integration time of the camera, *M* (*x*, *y*, *t*) denotes the modulation function between the DMD and CCD, *T* represents one frame integration time of CCD camera and point (*x*, *y*) represents an arbitrary coordination on the DMD corresponding to a pixel on the CCD sensor. Therefore, actually detected light intensity *V*(*x*, *y*) of the DMD camera can be expressed as

(5) $$V(x,y)=\sum_{t=0}^{T}M(x,y,t)\cdot I(x,y,t)=\sum_{t=0}^{T}M(x,y,t)[{\left|f(s,t)\right|}^{2}⊗{\left|h(x,y;s,t)\right|}^{2}],\;(0\leq V\leq 255)$$

where *M*(*x*, *y*, *t*)∈ [0, 1]. When *M*(*x*, *y*, *t*) = 0, all DMD micromirrors are closed, no light can reach the CCD camera; when *M*(*x*, *y*, *t*) = 1, all DMD micromirrors are opened. That is, for the traditional camera, *M*(*x*, *y*, *t*) = 1 ∀ (*x*, *y*, *t*).

### 2.3. The Correspondence Adjustment of DMD-to-CCD

The correspondence of DMD-to-CCD is the precondition to realize high precision modulation. In this section, we had achieved accurate mirror-to-pixel correspondence adjustment in DMD camera by using the phase-shifting moiré method based on the work of Ri *et al.* [32].

**Figure 3:**
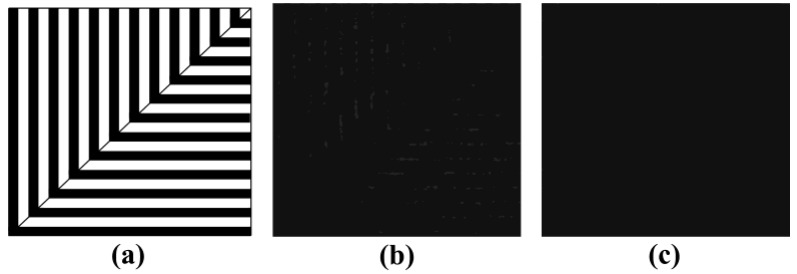
Result of correspondence of DMD-to-CCD. (**a**) Gating pattern taken by DMD camera; (**b**) Not corresponding; (**c**) Corresponding.

As we all know, a geometric moiré fringe pattern will appear when two similar grating strips with slope angle are superimposed. In our experiments, we had designed a calibration periodic grating pattern to analyze the *x* and *y* directional phases of the moiré fringe pattern simultaneously, as shown in Figure 3a. Each periodic grating pattern consists of four mirrors, two on and two off. Similarly, the pixels of CCD camera with every four pixels as one period are equal interval sampled. Then the first pixel in each period is selected as pixel sampling value and duplicated it to another three pixels to make all the pixels with the same value in each period. The initial result of correspondence of DMD-to-CCD is shown in Figure 3b, and the phase-shift moiré fringe phenomenon appears. The reason is that when the DMD and CCD are not corresponding, due to the sampling pixel values of neighboring periods are different, the grayscales of periodic stripe pattern are changed gradually and the moiré fringe can be easily observed after replication and interpolation. However, when each mirror of DMD and each pixel of CCD are corresponding, the image captured by CCD camera will not show a stripe pattern since all the pixels have the same value in each period. Therefore, the correspondence of DMD-to-CCD can be detected with high accuracy by using the presence or absence of moiré fringe pattern.

In our DMD camera, we adjusted the *xyz*-θ stage to change the positions of the CCD and lens 1 to realize the correspondence of each mirror of the DMD to each pixel of the CCD. Figure 3c shows the experimental results, and the phase-shifting moiré fringe gradually disappears with fine-tuning platform. That is, each DMD mirror corresponding to each CCD pixel with high accuracy is implemented.

## 3. The Theory of Per-Pixel Coded Exposure for High-Speed and High-Resolution Imaging

In the present section, we propose a theory of per-pixel coded exposure for high-speed and high-resolution imaging, which can accurately reveal the DMD pixel-level modulation process. Then we discuss the sampling clock control based on the clock features of DMD and CCD to achieve the synchronization. Finally, an extraction algorithm of subframe is proposed to combine different exposure pixels into a new subframe based on the light intensity distribution of the image.

### 3.1. Working Principle of Per-Pixel Coded Exposure

As mentioned above, the DMD camera belongs to secondary imaging system, and it can make sure that the images captured by CCD camera are obtained the DMD space-time modulation via the switching state and switching time of each DMD mirror. Also, because each DMD mirror corresponds to each CCD pixel, it means that mirrors and pixels are functionally equivalent. Thus the DMD micromirror array can be programmed to cycle through a series of binary coded patterns created on the image.

**Figure 4:**
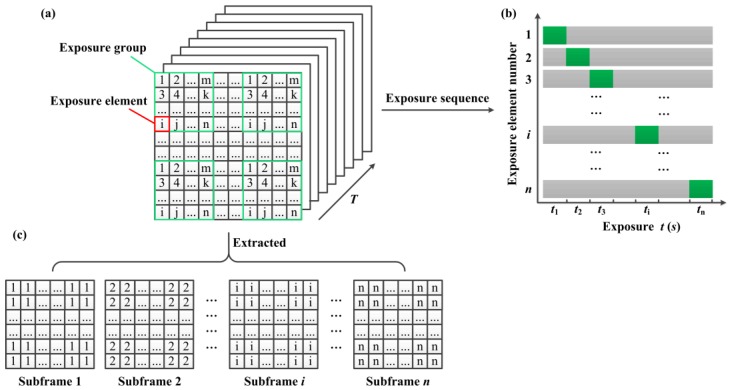
Working principle of per-pixel coded exposure. (**a**) DMD mask; (**b**) Exposure sequence for different exposure elements; (**c**) Subframes.

Individual mirrors on DMD plane are classified for programming purposes as being part of exposure elements, exposure groups, and subframe elements as shown in Figure 4a. Here, each DMD coded pattern can be taken as a mask. Suppose we subdivide a DMD mask into *m* non-overlapping exposure groups, each exposure group consists of *n* exposure elements. Each exposure element has the different identifiers in every exposure group but with the same identifier in all exposure groups. Figure 4b shows the individual exposure elements with same identifier are simultaneously exposed, and the exposure elements with different identifiers are sequentially exposed during one frame integration time of the CCD camera. So that at any instant, the grayscale of corresponding pixels in image sensor are different because of different exposure time. That is, the temporal information has been embedded in each frame by per-pixel coded exposure. The coded exposure images with full-resolution are read out and stored after one frame integration time of the CCD camera. In addition, *n* subframes can be extracted from the resulting coded exposure image in all *m* exposure groups based on the different light intensity distribution of the image. Then, pixels labeled as 1 in all exposure groups are extracted to constitute subframe 1 and pixels labeled as 2 in all exposure groups are extracted to constitute subframe 2, and so on, until the composition of the subframe *n* as shown in Figure 4c. Therefore, the coded exposure images with the full-resolution are unchanged at native detector frame and the spatial resolution of each subframe is *n* times lower than that of the coded exposure image, but the temporal resolution of the DMD camera can be greatly improved by *n* times higher than the conventional image-capture mode of the same detector. Thus, the per-pixel coded exposure for high-speed and high-resolution imaging by using a DMD camera can be implemented without increasing the inherent properties of camera.

Suppose we expect to increase the temporal resolution of imaging system by *N* times, *i.e.*, *N* frames different DMD masks have been sequentially exposed in one frame integration time of the CCD camera. Let *M_i_*(*x*, *y*, *t*) denote the DMD mask function, which is active for *t_i_* ms, and *M*’(*x*, *y*, *t*) represents the per-pixel coded exposure modulation function which can accurately reveal the DMD pixel-level modulation process can be expressed as

(6) $$M^{'}(x,y,t)=\sum_{i=1}^{N}M_{i}(x,y,t)t_{i}$$

Combined with the optical model of DMD camera, the actually detected light intensity *V*’(*x*, *y*) by using the per-pixel coded exposure method can be expressed as

(7) $$V^{'}(x,y)=\sum_{t=0}^{T}\sum_{i=1}^{N}M_{i}(x,y,t)t_{i}[{\left|f(s,t)\right|}^{2}⊗{\left|h(x,y;s,t)\right|}^{2}],\;(0\leq V^{'}\leq 255)$$

Theoretically, this approach can rapidly increase the temporal resolution of the imaging system by several times, or even hundreds of times without increasing bandwidth requirements of the camera. In addition, each DMD mask can be represented by binary encoding, and exposure groups may have different shapes, including diamond-shaped, square, rectangular, and other nonrectangular regions of interest.

### 3.2. Sampling Clock Control

In DMD camera, the direction of each mirror can be controlled by digital signals within 24 μs, which is approximately 1/1000 of a frame time of an ordinary CCD camera, and it means that different patterns on the DMD can be projected for many times during one frame integration time of the CCD camera. Firstly, we need to make the CCD and DMD synchronization. Suppose that the DMD patterns are set to cycle for *N* times sequentially, the sampling period of CCD camera can be expressed as

(8) $$T=t_{i}\cdot N+\Delta t$$

where *Δt* stands for the frame readout time. In general, its value is very small.

**Figure 5:**
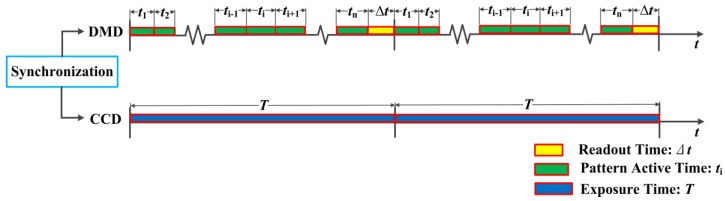
Sampling clock control of the DMD camera.

Figure 5 shows the sampling clock control of the DMD camera based on the clock features of CCD and DMD. The CCD camera is set to be triggered every *n* DMD patterns during the camera frame exposure time *T*. After that, frames are continually saved to hard disk for later analysis.

### 3.3. Extraction of Subframe

**Figure 6:**
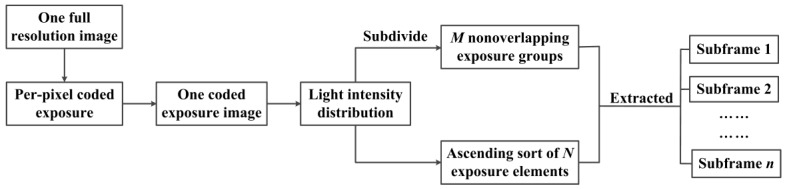
Flowchart of extraction of subframes.

The temporal information has been embedded into the coded exposure images captured by DMD camera through DMD per-pixel coded exposure modulation. We need to read the temporal information to extract the different subframes to achieve high-speed imaging. Generally, the grayscale of neighborhood pixels is smooth but now will appear a large difference because of the DMD modulation. Meanwhile, different exposure elements have the same sorting in their exposure groups, before or after the per-pixel coded exposure. Therefore, we can sort the exposure elements based on their ascending grayscale in each exposure group, and the resulting elements sequences can be labeled as 1, 2, 3, ..., *n*, which corresponds to the CCD pixels. Therefore, *n* frames subframes can be extracted from one coded exposure image, that is, the temporal resolution of DMD camera can be greatly improved by *n* times. The flowchart of extraction of subframes is shown in Figure 6.

It becomes the key step that the exposure elements in each exposure are ascending sorted, and the sorting algorithm will directly determine the efficiency of image processing. Many algorithms have been proposed to implement the data sorting, such as bubble sort, select sort, insertion sort, merge sort, and quicksort [33]. The quicksort based on divide-and-conquer is currently recognized as the best kind of internal sorting algorithm and widely used because of its simple structure and above average performance.

However, the traditional quicksort algorithm has the following disadvantages. On the one hand, if the first element in data sequence is selected as the pivot, the worst time complexity *O*(*n*^2^) will probably appear when grouped data are partly identical or partly in order. On the other hand, the subsequences will become smaller and smaller with the execution of the algorithm, and when the length of the subsequence reduces to a certain value, the speed of quicksort will not run as fast as other primary sorting methods. As the large amount of image data in our research needs to be processed, it is necessary to improve the efficiency of traditional quicksort.

In this paper, a three-element median quicksort method is proposed to overcome the shortcomings of traditional quicksort method. Suppose the *n* disorderly data can be expressed as {*d_i_*} = (*d_1_*, ...,*d_n_*), the new algorithm can be described as follows.

Step1: three elements *d_j_*, *d_k_*, *d_m_* are randomly selected in sequence {*d_i_*}, and compared with each other to take their median *d_i_* as the pivot. Then the pivot *d_i_* will be compared with other elements, and the current disorderly sequence will be divided into the left subinterval *L_d_* = (*d_1_*, ..., *d_i−1_*) and the right subinterval *R_d_* = (*d_i+1_*, ..., *d_n_*). All the data in *L_d_* should be less than or equal to the pivot *d_i_* while all the data in *R_d_* should be greater than the pivot *d_i_*. The pivot *d_i_* keeps the original position unchanged.

Step2: all the data in *L_d_* or *R_d_* will make a recursive call with the Step1 to quicksort each element, respectively; until the data length is less than *k*, then Step2 stops;

Step3: the remaining left and right subinterval are respectively used the insertion sort: the data would be inserted in an appropriate position by being compared with the previously sorted data, until insertion sorting of all data are completed.

**Figure 7:**
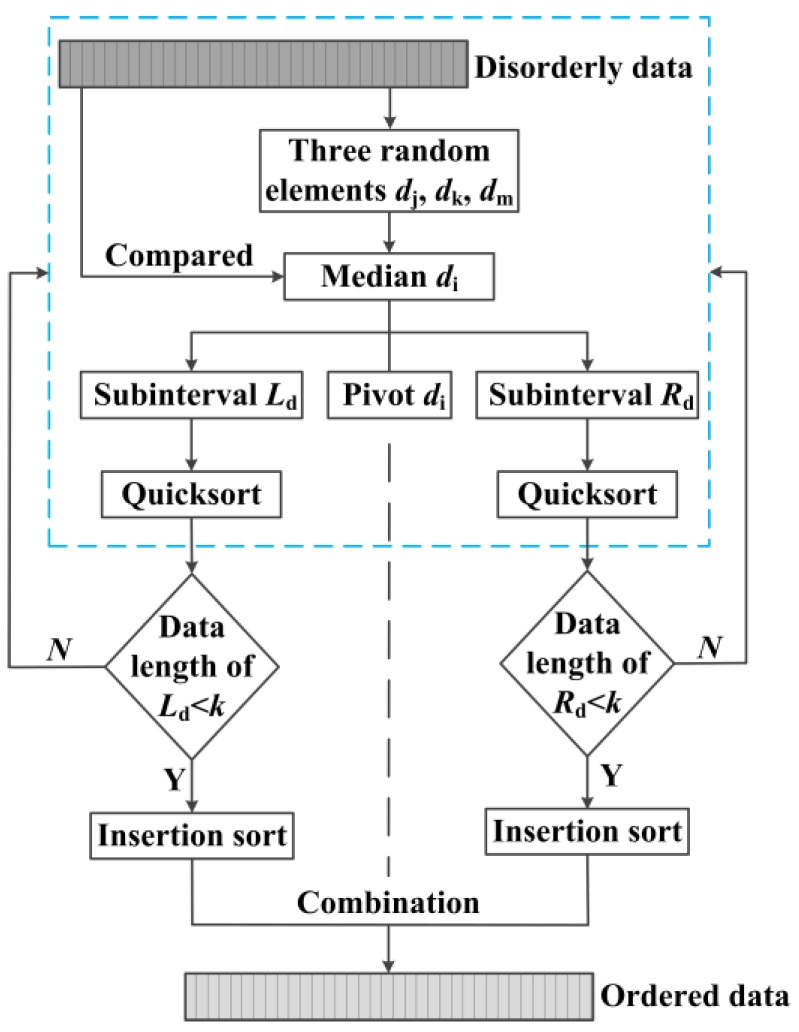
Flowchart of three-element median quicksort method.

Figure 7 shows the flowchart of three-element median quicksort method. After that, an ascending sequence of data will be available. In order to obtain the reasonable value of *k*, we need to calculate the time complexity of the new algorithm. The average time complexity of traditional quicksort can be expressed as

(9) $$T_{o}(n)=\frac{1}{n}\sum_{i=1}^{n}\left(T_{o}(i-1)+T_{o}(n-i)\right)+n\;(n>1)$$

where *T_o_*(*i −* 1) and *T_o_*(*n − i*) represent the average time complexity of subsequences *L_d_* and *R_d_*, respectively. When variable *i* changes from 1 to *n*, there will be two equal *T_o_*(0), *T_o_*(1), ..., *T_o_*(*n −* 1), so the Equation (9) is equivalent to

(10) $$T_{o}(n)=\frac{2}{n}\sum_{i=0}^{n-1}T_{o}(i)+n$$

The Equation (10) is respectively transposed and subtracted and we can get

(11) $$\frac{T_{o}(n)}{n+1}=\frac{T_{o}(n-1)}{n}+\frac{2n-1}{n(n+1)}$$

Then we define $u_{n}=\frac{T_{o}(n)}{n+1}$, the Equation (11) can be expressed as

(12) $$u_{n}=u_{n-1}+\frac{2n-1}{n(n+1)}$$

The recursive method is used in Equation (12), and it can be calculated as follows.

(13) $$u_{n}=2\sum_{i=2}^{n}\frac{1}{i}+\frac{3}{n+1}-\frac{3}{2}$$

Therefore, the average time complexity of traditional quicksort can also be expressed as

(14) $$T_{o}(n)=2(n+1)\sum_{i=2}^{n}\frac{1}{i}+3-\frac{3(n+1)}{2}\;(n>1)$$

Similarly, when the variable *i* changes from *k* to *n*, the average time complexity of quicksort can be expressed as

(15) $$T_{1}(n)=\frac{1}{n}\sum_{i=k+1}^{n}\left(T_{1}(i-1)+T_{1}(n-i)\right)+n=2(n+1)\sum_{i=k+1}^{n}\frac{1}{i}+3-\frac{3(n+1)}{k+1}\;(n>k)$$

The average time complexity of median comparison can be expressed as

(16) $$T_{2}(n)=n\;(n>1)$$

The average time complexity of insertion sort can be expressed as

(17) $$T_{3}(n)=\frac{n}{k}\frac{\left(k+4)(k-1\right)}{4}\leq \frac{nk}{4}+\frac{3n}{16}\;(1<n<k)$$

Therefore, the average time complexity of three-element median quicksort method can be expressed as

(18) $$T(n)=T_{1}(n)+T_{2}(n)+T_{3}(n)=2(n+1)\sum_{i=k+1}^{n}\frac{1}{i}+3-\frac{3(n+1)}{k+1}+\frac{nk}{4}+n\;(n>1)$$

Here, we define a new function *f* (*n*, *k*) to express the difference of the average time complexity of the above two method.

(19) $$f(n,k)=T_{o}(n)-T(n)=2(n+1)\sum_{i=2}^{k}\frac{1}{i}+3(n+1)(\frac{1}{k+1}-\frac{1}{2})-(\frac{k}{4}+1)n$$

Because of $\sum_{i=k+1}^{n}\frac{1}{i}\leq \int_{k}^{n}\frac{1}{x}dx=\ln n-\ln k=\ln\frac{n}{k}$, the Equation (19) can be expressed as

(20) $$f(n,k)=2(n+1)\ln k+3(n+1)(\frac{1}{k+1}-\frac{1}{2})-(\frac{k}{4}+1)n$$

When *f’*(*n*, *k*) = 0, the function *f* (*n*, *k*) will obtain extremum. Therefore, we define $\frac{\partial f}{\partial k}=0$, the Equation (20) can be expressed as

(21) $$\frac{2}{k}-\frac{3}{{\left(k+1\right)}^{2}}=\frac{n}{4(n+1)}\leq \frac{1}{4},\;(k\in N)$$

We calculate the inequality Equation (21) to get *k* ≥ 7, and the function *f* (*n*, *k*) obtains the maximum value, where the difference of the average time complexity of the above two methods is the maximum. Therefore, when the data length of left or right subinterval is less than 7, the insertion sort is used in the remaining left and right subinterval, and the average time complexity of three-element median quicksort algorithm has the minimum. In this case, the algorithm works best.

## 4. Experimental Results

In this section, we do experiments to use the method mentioned in Section 3 to capture the rapid phenomena with high-speed and high-resolution. In general, we can use the different shapes or different amount of DMD masks (such as 3 × 3, 9 × 9, 16 × 16 and other type grid patterns) to rapidly increase the temporal resolution of the imaging system by several times, or even hundreds of times. However, in our experiments, due to the limitations of our DMD resolution, we want to achieve four times gain in temporal resolution by using a 25 fps camera (*T* = 40 ms, *t_r_* = 0.2 ms), yielding a final frame rate of the embedded lower-resolution image sequence of 100 fps.

**Figure 8:**
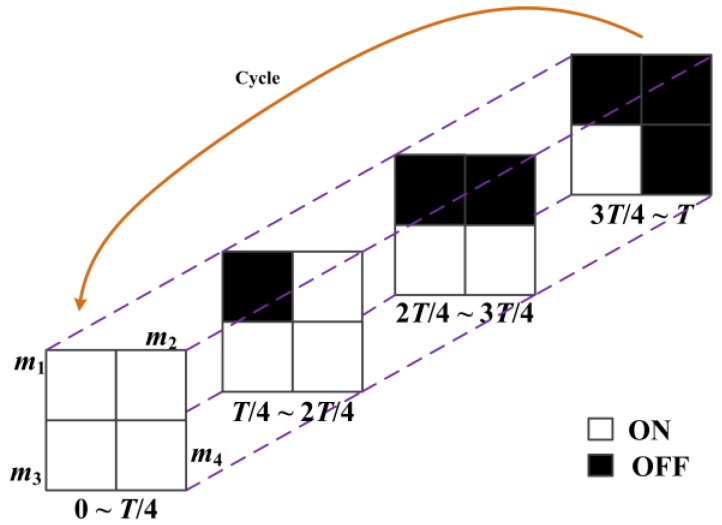
Modulation process of DMD mirrors in each exposure group.

Figure 8 shows the modulation process of DMD mirrors in each exposure group. The exposure group in DMD pattern mask consists of four (2 × 2 grid pattern) exposure elements, which are labeled as *m*_1_, *m*_2_, *m*_3_, *m*_4_, respectively. All the mirrors are opened during the exposure time from 0 to *T*/4; then *m*_1_ is closed and *m*_2_, *m*_3_, *m*_4_ are opened during the exposure time from *T*/4 to 2*T*/4; *m*_1_, *m*_2_ are closed and *m*_3_, *m*_4_ are opened during the exposure time from 2*T*/4 to 3*T*/4; finally, *m*_1_, *m*_2_, *m*_3_ are closed and *m*_4_ is opened during the exposure time from 3*T*/4 to *T*. The DMD pattern masks (active time *t_i_* = 9.8 ms) are set to cycle four times sequentially during a frame time of CCD camera. After that, one coded exposure image with full-resolution would be obtained and four different subframes could be extracted from the coded exposure image by using the three-element median quicksort method. In addition, all our experiments are done in a dark room and backlighted to highlight the phenomenon.

**Figure 9:**
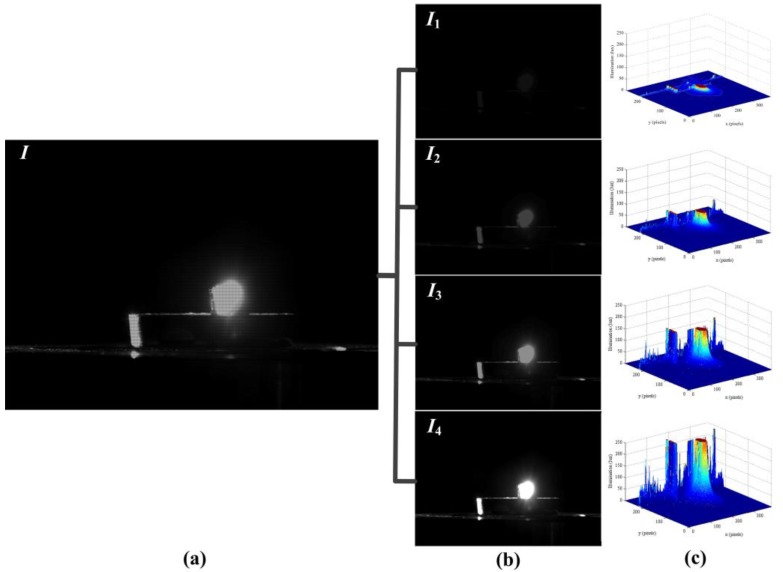
The first example result of coded exposure for the brightness changes of candle flame. (**a**) Coded exposure image *I*; (**b**) Subframes:*I*_1_, *I*_2_, *I*_3_, *I*_4_; (**c**) Light intensity of subframes.

**Figure 10:**
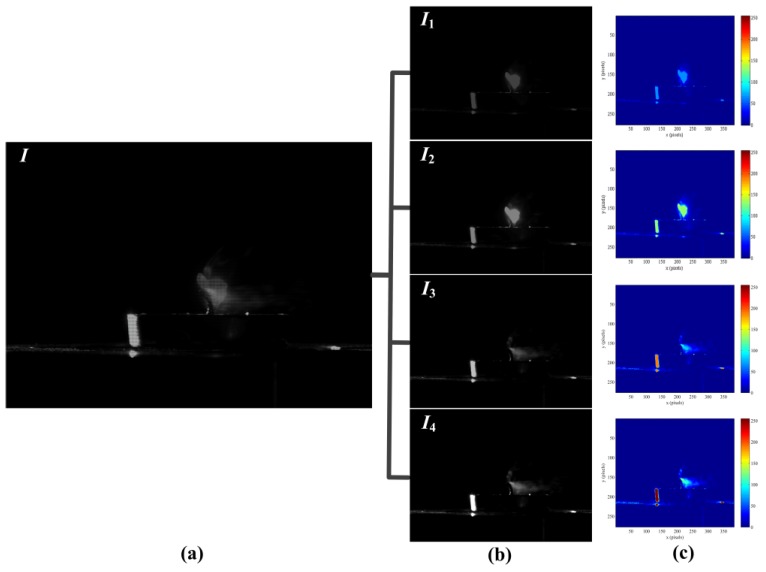
The second example result of coded exposure for the motion of the candles flame. (**a**) Coded exposure image *I*; (**b**) Subframes: *I*_1_, *I*_2_, *I*_3_, *I*_4_; (**c**) Light intensity distribution of candle flame.

**Figure 11:**
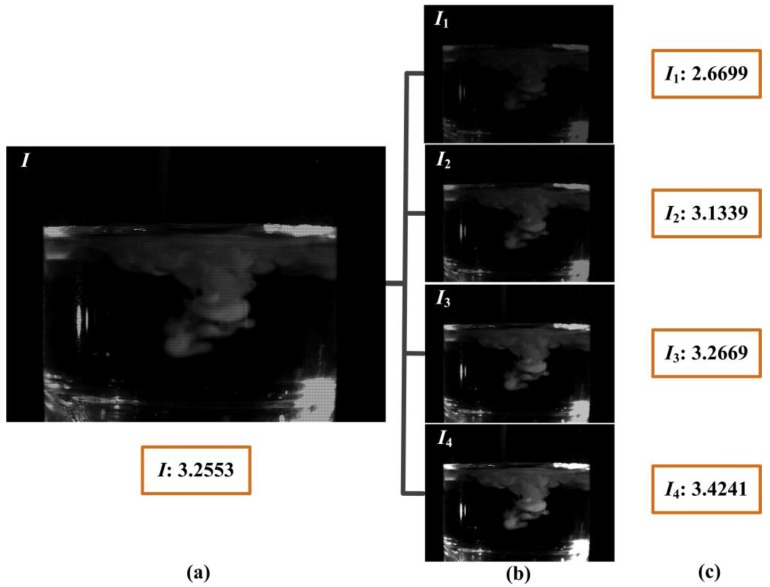
The third example result of coded exposure for subtle change of the liquid mixing motion. (**a**) Coded exposure image *I*; (**b**) Subframes: *I*_1_, *I*_2_, *I*_3_, *I*_4_; (**c**) Entropy of different frames.

We have used our DMD camera to capture different scenes comprising a wide range of high-speed images. The temporal information had been embedded into the coded exposure image with high-resolution (768 × 576 pixels, 25 fps), and the high-speed subframes (384 × 288 pixels, 100 fps) were recorded and decomposed the process of the high-speed imaging. In order to show the effect of the DMD camera for temporal information modulation, the first example demonstrated the brightness changes of candle flame as shown in Figure 9. The coded exposure image captured by DMD camera was shown in Figure 9a,b showed that four different subframes were extracted from Figure 9a. The light intensity of the subframes were increased gradually as shown in Figure 9c, thus it indicated that the candle flame was brighter with the growth of the exposure time. In a sense, increasing the temporal resolution is equivalent to make the fast-moving phenomenon gradually slow. Figure 10 recorded the second example result for the motion of the candles flame. Figure 10a was the coded exposure image which recorded the instantaneous change of blowing out the candles, and four subframes were extracted as shown in Figure 10b. Figure 10c showed that the light intensity distribution of candle flame was gradually decreased, so the whole process of blowing out candles was fully described. The subtle change of the liquid mixing motion was clearly shown in Figure 11. Figure 11a was the turbid coded exposure image, and Figure 11b recorded the process with four subframes when the milk was poured into the water. The entropy of image can represent the aggregation feature of image gray distribution, and the large entropy means that the image has more valuable. Figure 11c showed the entropy of different frames, the entropy of subframe *I*_3_ was very close to the coded exposure image *I*, and the entropy of subframe *I*_4_ was exceeded to the coded image *I*. That is, subframes with effective information had been gradually increased and the sharpness change of liquid mixing was accurately recorded. Therefore, all the experiments show that our method has achieved a good visual result for high-speed image.

## 5. Conclusions

In this paper, we propose an efficient way to capture high-speed and high-resolution images by using a DMD camera with per-pixel coded exposure method. Compared with the conventional high-speed imaging methods, we break through the bandwidth limitations of traditional cameras, design and build a DMD camera that could greatly increase the temporal resolution of the imaging system while increasing neither memory requirements nor the intrinsic frame rate of the camera. In order to overcome the disadvantages of the pixel binning, we use the optical model of the DMD camera, theoretically analyze the per-pixel coded exposure, and propose a three-element median quicksort method to realize the quick sorting of pixels. Theoretically, this approach can rapidly increase the temporal resolution of the imaging system several, or even hundreds, of times. In addition, this approach uses a new programmable imaging system, and it also supports different spatial light modulators or different frame rates for the different areas of the image sensor, which may be useful for unconventional detectors. Finally, we have achieved 100 fps gain in temporal resolution by using a 25 fps camera, and all experiments on a wide range of scenes show that our method has achieved a good visual result for high-speed images.

However, the proposed method has several limitations. Firstly, the physical resolution of DMD determines the maximum resolution of the DMD camera, especially maximum frame rate and maximum spatial resolution. Secondly, a DMD camera with low light efficiency cannot obtain high quality images in low-light conditions. For the future application of this technology, it is useful in various fields such as observing the activities of cells in the biomedical field, high dynamic range imaging, extending the depth of field photography, three-dimensional shape measurement, and so on, and it will encourage researchers and engineers to do further research and development.
